# Utilization and Evaluation of Rice Bran and Rice Bran Wax as a Tablet Lubricant

**DOI:** 10.3390/pharmaceutics16030428

**Published:** 2024-03-20

**Authors:** Ornanong S. Kittipongpatana, Karnkamol Trisopon, Phanphen Wattanaarsakit, Nisit Kittipongpatana

**Affiliations:** 1 Department of Pharmaceutical Sciences, Faculty of Pharmacy, Chiang Mai University, Chiang Mai 50200, Thailand; ornanong.kit@cmu.ac.th (O.S.K.); karnkamol.t@cmu.ac.th (K.T.); 2 Lanna Rice Research Center, Chiang Mai University, Chiang Mai 50100, Thailand; 3 Department of Pharmaceutics and Industrial Pharmacy, Faculty of Pharmaceutical Science, Chulalongkorn University, Bangkok 10330, Thailand; phanphen.a@chula.ac.th

**Keywords:** lubricant, rice bran, rice bran wax, pharmaceutical excipient, direct compression

## Abstract

The rice bran and rice bran wax of the KJ CMU107 rice strain were investigated as potential tablet lubricants in a directly compressed tablet formulation. Stabilized full-fatted rice bran (sFFRB), stabilized defatted rice bran (sDFRB), and rice bran wax (RBW) extracted and purified from crude rice bran oil (cRBO) were tested. Two commercial lubricants, including magnesium stearate (MGS) and hydrogenated cottonseed oil (HVO), were employed as the standards in the formulated mixtures, which contained spray-dried rice starch (SDRS) as a diluent. The tableting was carried out for each formulation, and the obtained tablets were physically and mechanically evaluated. Among the parameters investigated were the general appearance, ejection force, weight variation, hardness, friability, and disintegration time. The powder flow was also determined for each formulation. The results showed that the tablet ejection forces for all the lubricated formulations (58–259 N) were significantly lower than that of the non-lubricated control formulation (349 N). The use of sFFRB as a lubricant at 0.5–2.0% *w*/*w* could lower the ejection force up to 78%, but the hardness reduced so drastically that the formulations failed the friability test due to the chipping of the tablets’ edges. Moreover, sDFRB performed significantly better as the use at 0.5–1.0% *w*/*w* in the formulation helped to lower the ejection forces by up to 80% while maintaining the changes in the tablet hardness within 10%. RBW functioned effectively as a tablet lubricant at a concentration of 0.5% *w*/*w*, yielding tablets with good strength comparable to standard HVO lubricant while helping to reduce the ejection force by 82%. In formulations with good lubrication, i.e., friability < 1%, the powder flow was improved, and the tablet disintegration times were within the same range as the control and HVO formulations. In conclusion, sDFRB displayed a lubricant property at concentrations between 0.5 and 1.0% *w*/*w*, with slightly negative effects on the tablet hardness. RBW from KJ CMU107 rice was an effective tablet lubricant at 0.5% *w*/*w*, with no effect on tablet hardness. Both materials can be further developed for use as commercial lubricants in direct compression.

## 1. Introduction

Lubricants are one of the most important and indispensable excipients that play a pivotal role in the efficient manufacturing of pharmaceutical tablets. During tablet compression, the powder mixture, which typically contains the active ingredient(s), filler/diluents, disintegrants, and binders, among others, is compacted under high pressure, which can result in friction between the tablet surface and the punch surface and die cavities [1]. Without the presence of a lubricant in the formulation, this friction can lead to the sticking and picking of the tablets, which can compromise the quality of the tablet and the efficiency of the manufacturing process [2]. Lubricants help the powder mixture flow smoothly through the compression machine, prevent its adhesion to the surfaces, and reduce the wear and tear of the equipment. They also contribute to ensuring uniformity in tablet weight and hardness, which are important parameters in the quality control of the finished product. Commonly used tablet lubricants include magnesium stearate (MGS), stearate acid, sodium lauryl sulfate (SLS), and poloxamers [3], while glidants and flow enhancers, such as talcum and colloidal silicon dioxide, at suitable specifications and concentrations, were also reported to have lubrication properties [4,5]. However, some of these conventional lubricants possess disadvantages. MGS, for example, can negatively affect the tensile strength, disintegration, and dissolution of tablets, thus compromising the bioavailability and efficacy of the drugs [6,7,8]. Stearic acid can cause the tablets to become sticky and difficult to handle during the manufacturing process. Talcum has a concern over health hazards due to the presence of asbestos fibers in some natural talc deposits, while the role of SLS as a tablet lubricant has recently been questioned, with the experimental data not supporting its use in tablet formulations [9]. Other commercially available pharmaceutical lubricants include sodium stearyl fumarate, glyceryl dibehenate, L-leucine, and hydrogenated plant seed oils. There are also several new tablet lubricants being studied and developed, including alternative metallic stearates, such as sodium and calcium stearates [10] and hexagonal boron nitride [11].

The ever-growing pharmaceutical and nutraceutical industries have seen a steady increase in the use of the currently available excipients, as well as the need for the research and development of new materials to accommodate new processes and to broaden the range of products. With attention being given not only to effectiveness and safety but also to the aspect of environmental sustainability [12], many pharmaceutical and nutraceutical manufacturing companies have currently shown interest in using natural or naturally derived excipients in their production processes. It is therefore important to develop new excipients, including lubricants, based on natural materials to meet such demand.

Rice bran is a part of rice grain that is taken off during the rice production process. It generally accounts for 8–10% of paddy weight [13,14,15]. Full-fatted rice bran (FFRB) is composed of carbohydrates (50%), oil (20–25%), and other health-benefited components, such as proteins, minerals, vitamins and derivatives, and dietary fibers. After the oil is extracted, the remaining marc, also known as defatted rice bran (DFRB) or rice bran meal, is usually discarded or sold off to animal farms as feedstock. Several studies have reported on the use of rice bran as an ingredient in animal feed and in human food products, such as cereals, bread, and snacks [16]. It has also been studied for its potential use in various industrial applications, such as in the production of biofuels, bioplastics, and nutraceuticals [17,18,19]. The use of rice starch as a pharmaceutical excipient has long been established. Rice husk, of which the main component is silicon dioxide, has been investigated as a potential glidant and lubricant [20]. Although less studied, rice bran possesses great potential due to its availability, sustainability, nutritional and other beneficial properties, and value-added possibilities [21]. The wax extracted from rice bran (RBW), which is composed of long-chain C22, C24, and C34 fatty acid esters, C18–C34 fatty alcohols, and C16–C26 fatty acids [22], was considered a novel excipient for the pharmaceutical dosage form [23]. It has been employed as an ointment base [24,25] and has successfully been used as a natural lipid composition for solid lipid nanoparticle (SLN) delivery systems [26]. However, neither FFRB nor DFRB have been investigated as tablet excipients, particularly as a lubricant, despite their potential, as suggested by their lipid compositions. This is partly due to the problems of the oxidation and rancidity of the material triggered by endogenous enzymes. To circumvent these problems, the stabilization of rice bran is recommended. Stabilized rice bran (sRB) can be produced by, among many other methods, heating the rice bran materials via microwave treatment [14] or steam sterilization to inactivate the enzymes that play a major role in the rancidity of rice bran due to oxidation [15].

As part of a research study to gauge the potential and optimize the use of rice bran and its compositions as different types of excipients in pharmaceutical formulations, this study investigated the physicochemical properties of the natural and stabilized rice bran samples and isolated rice bran wax as well as their pharmaceutical functionality as tablet lubricants.

## 2. Materials and Methods

### 2.1. Materials

Rice bran of the Khum Jao Mor Chor 107 (KJ CMU107) rice strain was provided by the Lanna Rice Research Center, Chiang Mai University, Chiang Mai, Thailand, in the form of full-fatted rice bran (FFRB) powder. The spray-dried rice starch (SDRS, Era-Tab^®^) was supplied by the Erawan Pharmaceutical Research and Laboratory (Bangkok, Thailand). The magnesium stearate was from Union Science Chemical (Chiang Mai, Thailand). The hydrogenated cottonseed oil (HVO, Lubritab^®^) was a product of JRS Pharma (Rosenberg, Germany). A commercial-grade rice bran wax, NatureSoft 860 GMP, was a gift from Micro Powders, Inc. (Tarrytown, NY, USA). All the solvents used in this study were of analytical grade.

### 2.2. Acquisition of the Crude Rice Bran Oil and the Preparation of the Defatted Rice Bran Samples

The FFRB powder was subjected to a cold-pressing procedure using an AS2000 cold-press machine (Alangkarn Siam, Nakhon Pathom, Thailand) to separate the crude rice bran oil (cRBO) from the powder. The remaining marc, called cold-pressed rice bran (CPRB), was collected as thin brittle plates, weighed, ground into a powder, and passed through sieve #40. One hundred (100) grams of this CPRB was then extracted with hexane using a Soxhlet apparatus for 3 h to obtain defatted rice bran (DFRB).

### 2.3. Isolation and Purification of the Rice Bran Wax

The isolation of the rice bran wax was carried out using the winterization technique. In brief, 20 g of cRBO was suspended in 100 mL of hexane, heated to 60 °C, and filtered. The solution was allowed to cool to room temperature, then was kept at temperatures of 18, 16, 14, and 12 °C for 1 h at each temperature. Finally, the mixture was kept at 10 °C overnight, followed by centrifugation at 10,000× *g* for 20 min. For purification, the crude rice bran wax (cRBW), which precipitated at the bottom of the container, was collected and redissolved in isooctane, heated to 80 °C, allowed to cool, and then re-centrifuged at 10,000× *g* for 20 min. Finally, the RBW was collected, powdered, and subjected to physicochemical studies.

### 2.4. Physicochemical Properties of the RBW Sample

The solubility, melting point, specific gravity, saponification, acid, and iodine values were determined for the obtained RBW samples. The solubility was tested in water and organic solvents, and the melting point was determined according to the standard USP method.

The saponification value, which is the mass in milligrams of KOH required to neutralize the free fatty acids and saponify the esters contained in one gram of sample, was determined according to the standard USP method [27] with slight modifications. RBW (10,000 g) was placed into a 100-mL flask containing 20.0 mL of the ethanolic potassium hydroxide solution. The mixture was heated under reflux conditions for 1 h, then titrated with 0.5 M HCl using a phenolphthalein solution as the indicator. A control experiment was carried out similarly but without the RBW. The saponification value was calculated using Equation (1), where 56.11 is the molecular weight of KOH, V_1_ and V_2_ are the titration volumes (mL) of the RBW sample and the control, respectively, and W is the weight of the sample (g).

Saponification Value = (56.11 × (V_1_ − V_2_))/W (1)

The acid value, which is the mass in milligrams of KOH needed to neutralize the fatty acids in one gram of sample, was analyzed using the standard USP method [27] with slight modifications. RBW (10,000 g) was dispersed in 5 mL of a 95% ethanol–petroleum ether (1:1) mixture and heated until it dissolved. A few drops of a phenolphthalein solution were added, and the mixture was titrated with 0.1 N KOH to the pink color end-point. The acid value was calculated using Equation (2), where 56.11 is the molecular weight of KOH, N is the normality of KOH, V is the titration volumes (mL), and W is the weight of the sample (g).

Acid Value = 56.11 × V × (N/W)(2)

The iodine value, which is the mass in grams of iodine that reacts with 100 g of sample, was determined using the Hanus method described in USP [27]. The RBW (10,000 g) was weighed in a 250-mL iodine flask and dissolved in 10 mL of chloroform. Slowly, we added 25.0 mL of iodobromide TS. The flask was stoppered and kept in the dark for 30 min with occasional shaking. Potassium iodide TS (30 mL) and water (100 mL) were added to convert the unreacted iodobromide into iodine. A titration was then carried out with 0.1 N sodium thiosulfate VS with vigorous shaking. When the iodine color faded, we added 3 mL of starch TS and continued the titration by adding the 0.1 M sodium thiosulfate solution drop-wise until the blue color disappeared. A blank test under the same condition was performed, and the value was recorded for calculation using Equation (3), where 126.90 is the atomic weight of iodine and V_B_ and V_S_ are the volumes (mL) of 0.1 N sodium thiosulfate used in the blank and sample tests, respectively. N is the normality of the sodium thiosulfate VS, and W is the weight (g) of the sample.

Iodine Value = (126.90 (V_B_ − V_S_) N)/10 W (3)

### 2.5. Scanning Electron Microscopic (SEM) Analysis

The SEM experiments, which were used to analyze the granule surface, shape, and size, were conducted using a TESCAN instrument, model CLARA (TESCAN, Brno, Czech Republic). The acceleration voltage was 10 keV. The sample was placed on a copper stub covered with adhesive tape and coated with gold under vacuum using a safematic CCU-010 coating system (safematic GmbH, Zizers, Switzerland). The images were taken at 3000× magnification.

### 2.6. Stabilization of the Rice Bran Samples

Three rice bran samples, FFRB, CPRB, and DFRB, were analyzed using the method described by Espinales et al. [28], with slight modifications. Fifty (50) grams of the sample was placed in a stainless steel can, steam-heated at 121 °C for 20 min, allowed to air-dry overnight, then dried in a hot-air oven at 60 °C for 24 h. The obtained powder samples, namely sFFRB, sCPRB, and sDFRB, were passed through sieve no. 40 and stored in polyethylene bags at 28 ± 2 °C until they were used.

### 2.7. Proximate Analyses

The analyses of moisture content, ash, and protein and fat in the samples were carried out according to the AOAC 925.19 [29], AOAC 942.05 [30], AOAC 992.23 [31], and AOAC 920.39 [32] standards, respectively.

### 2.8. Powder Flow

The flowability of the powder samples used in the preparation of the tablets was evaluated through the determinations of the angle of repose (AR), compressibility index (CI), and Hausner ratio (HR) [33].

The angle of repose was determined by pouring the sample (100 g) through a glass funnel fixed at a height of 15 cm onto a level bench top. The height (h) and radius (r) of the formed conical pile were measured, and the tangent of the angle of repose was calculated using the h/r ratio according to Equation (4). The test was carried out in triplicates.

Tan θ = h/r(4)

The compressibility index (CI, %) and Hausner ratio (HR) were calculated from the bulk and tapped densities of the powder samples. In brief, 50 mL of powder was poured into a 100-mL graduated cylinder via a glass funnel, and its weight was recorded. The bulk and tapped densities were determined using Equations (5) and (6) as the ratio of the powder weight to the powder volume before tapping and after tapping using a jolting volumeter (Stampfvolumeter SVM, Erveka, Germany) until there were no further changes in volume. Carr’s index was the percentage ratio of the differences between the values of the two densities to that of the tapped density (Equation (7)), while the Hausner ratio was the ratio of the bulk density to the tapped density (Equation (8)).

ρ_bulk_ = m/V_bulk_ (5)

ρ_tapped_ = m/V_tapped_ (6)

CI = 100 × (ρ_tapped_ − ρ_bulk_)/ρ_tapped_ (7)

HR = ρ_tapped_/ρ_bulk_ (8)

### 2.9. Tabletability of the Rice Bran Powders and Tablet Compositions

The powder sample (250 mg) was compressed using a hydraulic press at compression forces of 0.5, 1.0, and 2.0 Ton using 14.23-mm flat face punches. No lubricant was applied to the punch & die set in the process. The hardness of each compact was measured in triplicates using a hardness tester (Erweka, Langen, Germany). A tabletability profile was plotted between the compression force (MPa) and the hardness (N) for all the samples.

### 2.10. Compression Test and Tablet Evaluation

The tablet formulations were composed of spray-dried rice starch (SDRS) (Era-tab, Cho Heng Vermicelli Factory, Nakhon Pathom, Thailand) as a diluent (1000 g) and one of the standard or tested lubricants at 0.5–2% *w***/***w* concentrations. The controlled formulation contained SDRS with no lubricant. The SDRS was passed through sieve mesh#20. The lubricant was passed through sieve mesh#40 and then mixed with SDRS powder in a UECM-RD (Unity Equipment, Nonthaburi, Thailand) cube mixer for 5 min.

All of the tablets were produced using a Fette 102i Laboratory Tablet Press (Fette Compacting GmbH, Schwarzenbek, Germany) equipped with cylindrical round concave punches of 8 mm in diameter. The turret rotational speed was set at 13 rpm. The pre-compression and main compression forces were set at 0.4 and 4.5 kN, respectively. The tablet weight was set at 300 mg, with a tablet filling depth of 10.13 mm. The tablet cylinder height for the main compression and for the pre-compression were programmed at 2.70 and 6.00 mm, respectively. The speed of the feed wheel paddle was set at 10 rpm. The data, including the actual pre-compression force, actual main compression force, and ejection force, were collected after 10 min of operation. Any remarks regarding the appearance of the tablets were noted macroscopically. The surface and edges were also inspected microscopically under a stereo microscope. The finished tablets were subjected to determinations of weight variation, hardness, friability, and disintegration time according to standard USP methods.

### 2.11. Statistical Analyses

All of the tests were performed at least in triplicates, and the data were expressed as mean values. The statistical analyses were conducted using a one-way analysis of variance (ANOVA) in SPSS (version 19.0). The significance tests of the means were analyzed using Tukey’s honestly significant difference (HSD) multiple range test at a 95% confidence level (*p* < 0.05).

## 3. Results and Discussion

### 3.1. cRBO Yield

**Figure 1:**
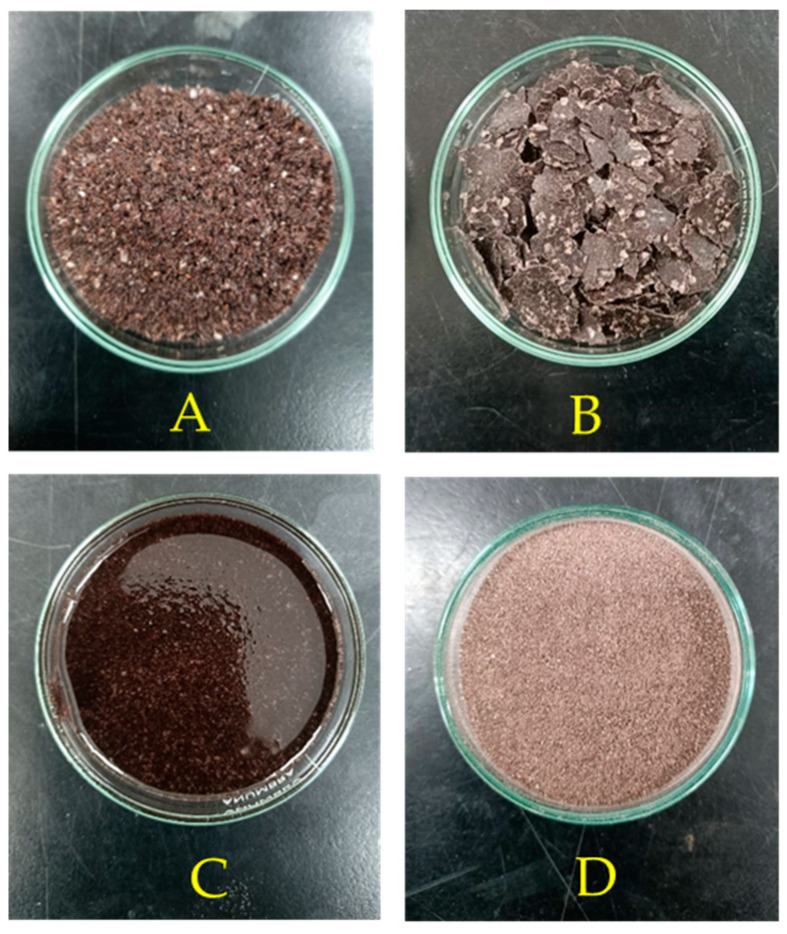
(**A**) Full-fatted rice bran (FFRB); (**B**) cold-pressed rice bran (CPRB); (**C**) crude rice bran oil (cRBO); (**D**) defatted rice bran (DFRB).

Upon subjecting the samples to the cold-pressing process, 1600 g of FFRB yielded 300 g (18.8%) of crude rice bran oil (cRBO) and 1300 g of CPRB (Figure 1). The oil yield was consistent with data that previously reported that rice bran contained 14–18% of crude oil [34]. The solvent extraction of 100 g of CPRB using hexane yielded DFRB and afforded 7.4 g of oily wax, which was subsequently combined with 23.1 g of cRBO and for use in the isolation and purification of the rice bran wax.

### 3.2. Isolation and Purification of Rice Bran Wax

**Figure 2:**
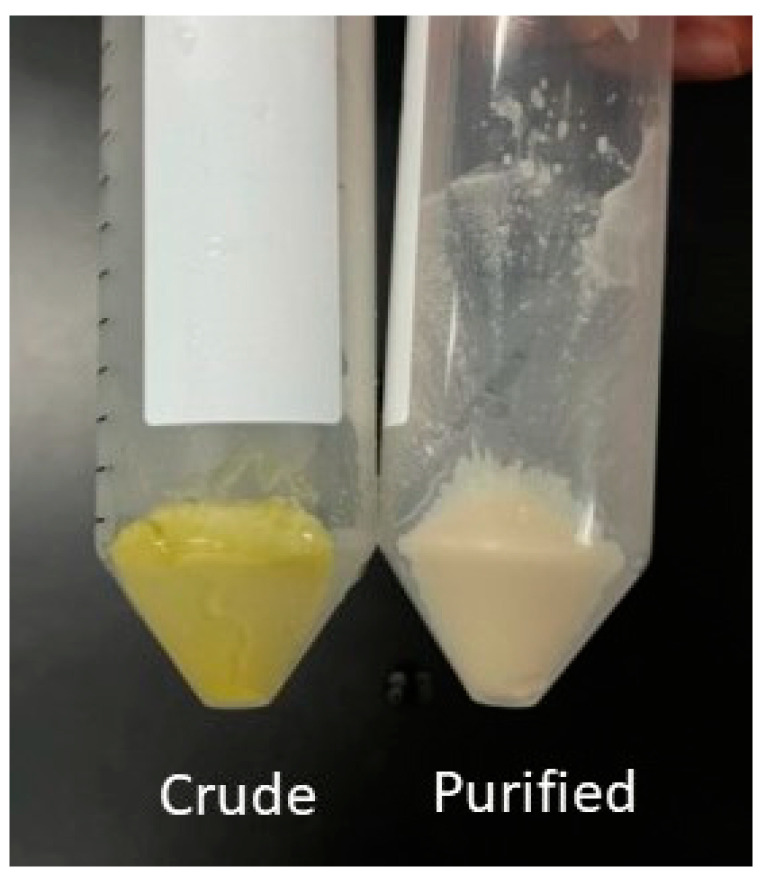
Crude RBW isolated from rice bran (**left**) and after purification (**right**).

The winterization of 30.5 g of cRBO yielded 2.01 g (6.6%) of the crude rice bran wax (cRBW). The wax samples were subjected to multiple washing steps until light-colored, purified rice bran wax (RBW) was obtained (Figure 2). The melting range of the isolated RBW (76–79 °C) was slightly lower than those reported previously [23,35]. The saponification, acid, and iodine values of 70.97 ± 5.59, 10.44 ± 0.52, and 10.28 ± 0.74, respectively, were similar to those of rice bran wax previously reported by Basarkar [23] (Table 1).

### 3.3. Stabilized Rice Bran Samples

The stabilization of the rice bran samples, carried out via thermal treatment with steam at 121 °C for 20 min, helped to decrease the oxidation and rancidity via the inactivation of enzymatic activity, including those of lipases, lipoxygenases, and peroxidases [14,15]. The appearances and textures of the stabilized samples, sFFRB, sCPRB, and sDFRB, were not significantly different from their native forms. The treatment conditions also sterilized the materials, thus extending their shelf life and allowing for the incorporation of biomaterials into tablet formulations.

### 3.4. Proximate Analysis of the Rice Bran Samples

As natural materials, it was very important that the quality control and analyses of the rice bran samples were carried out before they were used. The compositions and specifications, including the moisture, ash, and protein and fat contents, could vary widely depending on the sources and ages of the material, the preparation and analysis methods, and many other factors [15]. The removal of fat and oil from FFRB, for example, would change the proportion of the other components in the resulting DFRB sample [36]. In this study, the DFRB sample contained significantly lower moisture and fat contents, with higher ash and protein contents than those of FFRB and CPRB. The results were consistent with a previous report that highlighted that hexane defatting increased the protein content by removing the lipids [37], while an increase in ash content was likely a result of an increased proportion of fibers in the defatted materials. For the same rice bran samples, the stabilized powders possessed lower moisture contents (35–40%) and slightly but significantly higher protein contents (2–5%) than the native powders. The changes observed in the ash and fat contents between each pair were not significant (Table 2).

### 3.5. Scanning Electron Micrographs (SEM)

**Figure 3:**
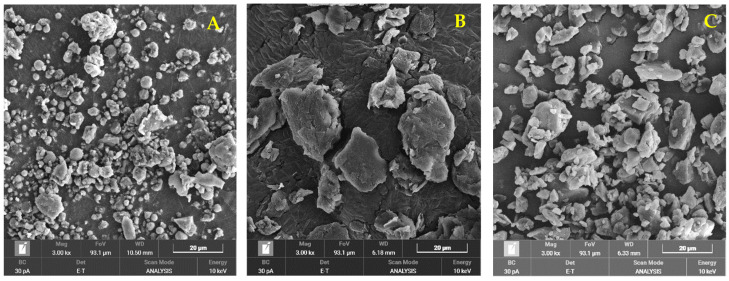
SEM images (3000×) of (**A**) full-fatted rice bran (FFRB); (**B**) KJ CMU107 rice bran wax (RBW); (**C**) commercial rice bran wax (NatureSoft 860 GMP).

The SEM images showed that FFRB contained particles of various sizes, ranging mostly from 2 to 6 μm, with some agglomerates and plate-like particles with a size of 10–12 μm. The small, spherical/polygonal particles were likely starch granules, while the larger plates and agglomerates were possibly fibers laminated with wax (Figure 3A). The RBW isolated and purified from the rice bran sample (Figure 3B) appeared as plate-like particles of 15–30 μm, with small, irregularly shaped debris. The debris shape and size (1–5 μm) were consistent with those of commercial rice bran wax (Figure 3C).

### 3.6. Powder Flow

The angle of repose (AR), compressibility index (CI) and Hausner ratio (HR) of the spray-dried rice starch (SDRS; F-1) were 32.2 ± 0.2, 11.9 ± 0.6, and 1.13 ± 0.01, respectively. These numbers, which indicated a “good” flow, were similar to those reported by Hsu et al. [38] (AR = 33.69; CI and HR = 13.33 and 1.15, respectively). The magnesium stearate and defatted rice bran exhibited “fair” mobility, while the values of full-fatted and cold-pressed rice bran samples indicated “poor” flow (Table 3). At the concentrations added into the formulations, all of the standards and some of the tested lubricants helped improve the powder flow; none had a negative effect.

### 3.7. Tabletability of the Rice Bran Powders and Tablet Compositions

**Figure 4:**
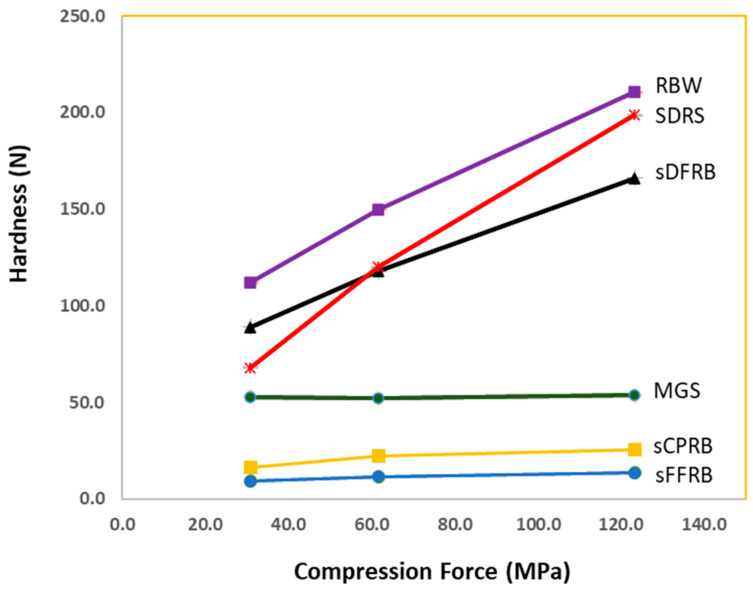
Tabletability profile of the materials in the tablet formulations.

The tabletability profile of sFFRS was very poor. The powder formed a very soft compact with low hardness when a compression force was applied through the hydraulic press. The surface of the compact was oily, suggesting that the oils inside the powder particles were forced out. The increase in the compact’s hardness was not achieved in response to increases in the compression force (Figure 4). A similar profile was observed for MGS, except the hardness of the MGS compact was significantly higher. The profiles of sDFRB and RWB, on the other hand, were satisfactory. The powders formed solid compacts, and the hardness increased proportionately with higher compression forces. SDRS, a direct compression excipient, exhibited very good tabletability but showed traces of particles sticking to the punch & die set.

### 3.8. Compression Test and Tablet Evaluation

The ejection force is a commonly used parameter to determine the lubrication ability and evaluate the lubricant’s overall effectiveness in tablet manufacturing. During tablet compression, the ejection force can be measured using a tablet press equipped with sensors that detect the force required for the lower punch to nudge the tablet out of the die [39]. The lower the ejection force, the better the lubrication, as it indicates that the tablet is moving smoothly through the die cavity. High ejection forces usually indicate inadequate lubrication, which coincides with one or more of the defects observed in the tablets, including chipping, sticking, picking, and binding [40]. The results showed that the control formulation (F-1), in which no lubricant was added, yielded mostly broken and chipped tablets, which indicated that the addition of a lubricant was necessary. At 0.5% *w*/*w* (F-4), sFFRB helped to decrease the ejection force by 25%, but the resulting tablets remained mostly broken, indicating inadequate lubrication. In the formulations containing sFFRB at 1.0 (F-5) and 2.0% *w*/*w* (F-6), the ejection forces required were further reduced to 25 and 22%, respectively, of the non-lubricated control. The resulting tablets, however, showed significant decreases in the hardness and did not pass the friability test. This was possibly due to the presence of sFFRB, which contained 12.8% fat in the formulation, which impeded the interparticulate bonding of SDRS. In contrast, the use of 0.5–2.0% *w*/*w* sDFRB as a tablet lubricant exhibited the ability to lower the ejection forces for the F-7, F-8, and F-9 formulations by 75–80% of the control value while compromising only up to 10% of tablet hardness. The fat content in sDFRB (3.52%) was much lower than that of sFFRB, as the materials had been defatted almost exhaustedly via solvent extraction, which led to a significantly better compaction as shown in the tabletability profile (Figure 4). The formulation containing 0.5% RBW (F-10) exhibited the lowest ejection force among the tested lubricants, with its reduction percentage (82%) lying between that of the two standard lubricants, MGS (F-2, 84%) and HVO (F-3, 76%), at the same concentration. The hardness of the F-10 tablets was comparable to that of the control, which indicated that the RBW did not affect or alter the tablets’ hardness. The friability was also within the 1% acceptable range (Table 4).

It is widely recommended that the evaluation of lubricant effectiveness via ejection force determination be used in conjunction with other methods to achieve optimal lubrication in all aspects of the specific tablet formulation and manufacturing process. These methods include the determination of tablet weight variation, hardness, friability, and disintegration time. In this study, the variation in the tablet weight was not significant among the different formulations. The tablets produced from all the formulations weighed around 300 mg as designed, with less than 1% deviation, which confirmed that the powder flow was sufficient. The omission of a glidant (e.g., talcum and silicon dioxide) from all the formulations was to ensure that the lubricating effect observed in the tests would be contributed solely by the tested lubricants, as some of the flow enhancers were known to possess lubrication properties [4,5].

**Figure 5:**
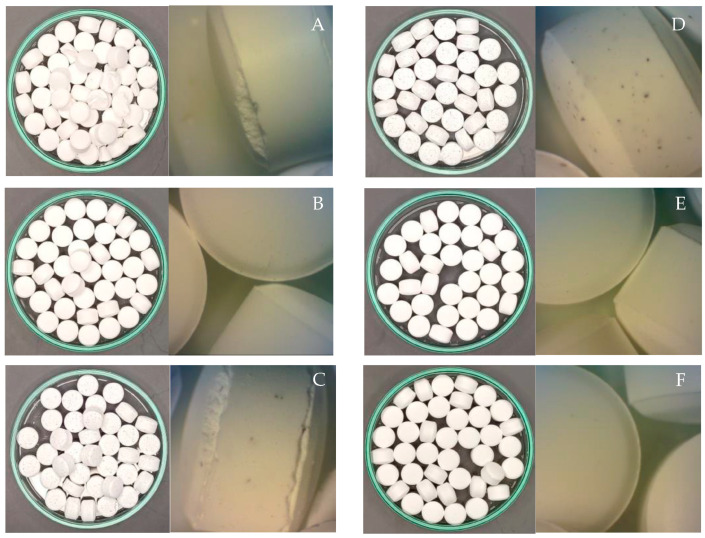
Macroscopic (**left**) and microscopic (**right**) appearances and remarks of the tablet formulations containing different lubricants. (**A**) Control (no lubricant); (**B**) 0.5% MGS; (**C**) 1.0% sFFRB; (**D**) 1.0% sDFRB; (**E**) 0.5% HVO; (**F**) 0.5% RBW.

The hardness and friability, in contrast, showed significant differences among the different tablet formulations. This was solely due to the type and concentration of the lubricant used since the formulations contained only SDRS and the lubricants. The hardness of the control formulation (F-1) was standard for the direct compression excipient, though many tablets appeared broken as they were produced without lubrication. The formulation containing 0.5% *w*/*w* MGS as a lubricant (F-2) yielded tablets with a hardness 23% less than that of F-1, but it did pass the friability test. The well-documented decrease in the tablet hardness when MGS was used as a lubricant was due to the formation of films on other tablet excipients during prolonged mixing [2]. The perfect appearance of the F-2 tablets was confirmed by examining the samples under a stereo microscope, which showed no defects on the surfaces or edges of the tablets (Figure 5). A similar observation was present for the F-3 tablets, which employed 0.5% *w*/*w* hydrogenated vegetable oil (HVO) as the lubricant, except that the hardness of the F-3 tablets was enhanced by the incorporation of HVO. For the tested lubricants, the formulations containing sFFRB failed the friability test at all three concentrations. This was likely a result of the decreases in the tablet hardness, in which the values of the F-4, F-5, and F-6 formulations were 17, 39, and 63%, respectively, which was lower than that of the F-1 tables. The tablets’ appearance under a microscope also revealed chipping on the edges, which indicated inadequate lubrication. Since a higher concentration in the formulation would likely further lower the tablet hardness, sFFRB was not a suitable lubricant, according to this study. In contrast, the F-7, F-8, and F-9 formulations, which contained sDDFB as a lubricant, yielded tablets with suitable hardness. Only F-9, which employed 2.0% *w*/*w* sDFRB and had a 10% decrease in hardness, failed the friability test. The formulations containing 0.5 and 1.0% *w*/*w* sDFRB showed good integrity and appearance both upon macroscopic and microscopic examinations. The results suggested that the optimum concentration of sDFRB as a lubricant was below 2.0% *w*/*w*. The use of RBW as a lubricant at 0.5% *w*/*w* in F-10 yielded tablets with suitable appearance and hardness, which was comparable to that of F-1. The tablets passed the friability test and appeared perfect under the microscope.

The disintegration times (DTs) of the tablet formulations containing sFFRB as the lubricant were shorter than those of the non-lubricated control tablets (Table 4). This was mostly due to the presence of the low-compactable sFFRB in the tablet texture, which contributed to the low tablet hardness and high friability. The other formulations showed slight or minimum increases for their DTs, except for one notable formulation containing 0.5% MGS, in which the DT was significantly prolonged. This is a well-known phenomenon when MGS is used as a lubricant due to the formation of MGS films on other components in the formulation [7,8].

## 4. Conclusions

Rice bran products in the forms of stabilized full-fat rice bran (sFFRB), stabilized defatted rice bran (sDFRB), and isolated rice bran wax (RBW) were prepared and tested as lubricants in the direct compression of spray-dried rice starch tablet formulations. All the formulations yielded tablets with good weight variation and lowered ejection forces compared to the non-lubricated control, but the formulations containing sFFRB showed drastic drops in tablet hardness as the concentration increased and failed the friability test. The use of sDFRB as a lubricant was effective at concentrations of 0.5–1.0% *w*/*w*, while at a higher concentration, the continued decrease in the ejection force was compromised by the lower tablet hardness and higher friability. The formulation containing 0.5% *w*/*w* RBW yielded tablets with comparable hardness to that of the control. The 82% ejection force reduction was comparable to that of 0.5% *w*/*w* MGS (83%) without prolonging the disintegration time. It could be concluded that the RBW isolated from KJ CMU 107 rice bran can be used as a tablet lubricant at the standard concentration, while sDFRB powders could potentially be an acceptable lubricant when used at higher concentrations. Further studies on sDFRB should include the investigation of the relationship between the fat content in the material and the lubricant activity and tabletability to establish the material specification to be used as a lubricant.

## Figures and Tables

**Table 1:** Physicochemical properties of rice bran wax isolated from this study in comparison with those previously reported.

Properties	This Study	Basakar [23]	Maru et al. [35]
Melting range (°C)	76–79	78–84	80.5
Solubility	Insoluble in water and soluble in EtOH, iso-PrOH, hexane, and isooctane	Insoluble in acetone and water and soluble in chloroform and petroleum ether	Insoluble in water and soluble in ether, EtOH, and iso-PrOH
Specific gravity	0.89 ± 0.05	0.93	0.912
Saponification value	70.97 ± 5.59	68–72	80.88
Acid value	10.44 ± 0.52	12–14	2.848
Iodine value	10.28 ± 0.74	9–11	10

**Table 2:** Proximate analysis of rice bran and the stabilized rice bran samples.

Sample *	Moisture (g/100 g)	Ash (g/100 g)	Protein (g/100 g)	Fat (g/100 g)
FFRB	10.30 ± 0.11 ^a^	9.44 ± 0.26 ^b^	15.94 ± 0.15 ^c^	12.46 ± 0.24 ^a^
CPRB	9.33 ± 0.25 ^b^	6.95 ± 0.29 ^d^	14.61 ± 0.05 ^e^	11.04 ± 0.05 ^b^
DFRB	4.16 ± 0.11 ^d^	10.42 ± 0.36 ^a^	16.72 ± 0.18 ^b^	3.37 ± 0.07 ^c^
sFFRB	6.01 ± 0.12 ^c^	9.90 ± 0.15 ^b^	16.70 ± 0.10 ^b^	12.80 ± 0.07 ^a^
sCPRB	6.07 ± 0.04 ^c^	7.77 ± 0.39 ^c^	14.91 ± 0.07 ^d^	11.44 ± 0.19 ^b^
sDFRB	2.81 ± 0.09 ^e^	11.03 ± 0.31 ^a^	17.41 ± 0.25 ^a^	3.52 ± 0.34 ^c^

* FFRB: full-fatted rice bran; CPRB: cold-pressed rice bran; DFRB: defatted rice bran; sFFRB: stabilized full-fatted rice bran; sCPRB: stabilized cold-pressed rice bran; sDFRB: stabilized defatted rice bran. The values are the average ± standard deviation values. In each column, different superscripts indicate the statistical differences of the values (*p* < 0.05).

**Table 3:** Powder flow parameters of the excipient materials and formulations.

#	Sample	Angle of Repose	Carr’s Index	Hausner Ratio
RB-1	sFFRB	40.1 ± 0.5 ^a^	29.0 ± 1.0 ^a^	1.41 ± 0.02 ^a^
RB-2	sCPRB	38.2 ± 1.4 ^b^	24.7 ± 1.2 ^b^	1.33 ± 0.02 ^b^
RB-3	sDFRB	35.6 ± 1.0 ^c^	20.9 ± 2.4 ^c^	1.27 ± 0.04 ^c^
STD	MGS	37.6 ± 0.8 ^b^	18.5 ± 1.4 ^c^	1.23 ± 0.02 ^c^
F-1	SDRS	32.2 ± 0.2 ^d^	11.9 ± 0.6 ^fg^	1.13 ± 0.01 ^fg^
F-2	SDRS + 0.5% MGS	31.0 ± 0.4 ^e^	10.7 ± 0.6 ^h^	1.12 ± 0.01 ^g^
F-2	SDRS + 0.5% HVO	30.8 ± 0.4 ^e^	10.4 ± 0.5 ^h^	1.12 ± 0.01 ^g^
F-4	SDRS + 0.5% sFFRB	31.7 ± 0.3 ^de^	12.0 ± 0.9 ^f^	1.14 ± 0.01 ^f^
F-5	SDRS + 1.0% sFFRB	31.4 ± 0.1 ^e^	15.7 ± 0.5 ^d^	1.19 ± 0.01 ^d^
F-6	SDRS + 2.0% sFFRB	32.6 ± 0.4 ^d^	12.7 ± 1.2 ^f^	1.15 ± 0.02 ^ef^
F-7	SDRS + 0.5% sDFRB	31.6 ± 0.3 ^de^	14.0 ± 0.1 ^e^	1.16 ± 0.00 ^e^
F-8	SDRS + 1.0% sDFRB	31.0 ± 0.5 ^e^	14.7 ± 1.2 ^de^	1.17 ± 0.02 ^d^
F-9	SDRS + 2.0% sDFRB	31.4 ± 0.9 ^de^	14.3 ± 0.6 ^e^	1.17 ± 0.01 ^de^
F-10	SDRS + 0.5% RBW	31.0 ± 0.1 ^e^	11.0 ± 1.0 ^gh^	1.12 ± 0.01 ^g^

sFFRB: stabilized full-fatted rice bran; sCPRB: stabilized cold-pressed rice bran; sDFRB: stabilized defatted rice bran; SDRS: spray-dried rice starch (Era-Tab^®^); MGS: magnesium stearate; HVO: hydrogenated vegetable (cottonseed) oil. The values are the average ± standard deviation values. In each column, the different superscripts indicate the statistical differences of the values (*p* < 0.05).

**Table 4:** Ejection force (N) and QC parameters of the tablet formulations containing various tested lubricants.

#	Lubricant * (%)	PCF (kN)	MCF (kN)	Ejection Force (N)	Weight (mg)	Hardness (N)	Friability (%)	DT (s)
F-1	Control	0.4	4.3	349.67 ± 27.72 ^a^	302.4 ± 0.6	72.7 ± 3.4 ^b^	>10	38.55
F-2	MGS 0.5%	0.4	4.7	58.16 ± 5.54 ^i^	303.1 ± 0.8	56.3 ± 5.6 ^e^	0.16	55.13
F-2	HVO 0.5%	0.4	5.1	83.11 ± 5.22 ^e^	300.6 ± 1.0	85.0 ± 4.0 ^a^	0.13	38.75
F-4	sFFRB 0.5%	0.4	5.1	259.40 ± 16.56 ^b^	302.6 ± 1.0	60.3 ± 9.1 ^d^	1.72	36.28
F-5	sFFRB 1.0%	0.4	4.6	88.17 ± 5.80 ^d^	302.4 ± 1.1	44.7 ± 4.8 ^f^	4.26	29.02
F-6	sFFRB 2.0%	0.4	5.0	78.60 ± 5.88 ^f^	300.2 ± 0.6	27.1 ± 6.5 ^g^	6.89	27.22
F-7	sDFRB 0.5%	0.4	4.5	89.88 ± 5.43 ^c^	300.1 ± 0.7	71.1 ± 5.8 ^b^	0.46	43.56
F-8	sDFRB 1.0%	0.4	4.9	78.92 ± 4.66 ^f^	299.5 ± 0.8	66.2 ± 6.5 ^c^	0.89	42.05
F-9	sDFRB 2.0%	0.4	4.3	71.88 ± 1.20 ^g^	299.7 ± 0.9	65.8 ± 4.7 ^c^	1.44	35.36
F-10	RBW 0.5%	0.4	4.4	65.08 ± 3.32 ^h^	300.3 ± 1.1	74.7 ± 7.2 ^b^	0.39	41.60

* Control: no lubricant; MGS: magnesium stearate; HVO: hydrogenated vegetable oil (Lubritab); sFFRB: stabilized full-fatted rice bran; sDFRB: stabilized defatted rice bran; RBW: rice bran wax; MCF: main compression force; PCF: pre-compression force; DT: disintegration time. The values are the average ± standard deviation values. In each column, the different superscripts indicate the statistical differences of the values (*p* < 0.05).

## Data Availability

The raw data supporting the conclusions of this article will be made available by the authors on request.

## References

[1] Li J., Wu Y. (2014). Lubricants in Pharmaceutical Solid Dosage Forms. Lubricants.

[2] Morin G., Briens L. (2013). The Effect of Lubricants on Powder Flowability for Pharmaceutical Application. AAPS PharmSciTech.

[3] Desai D., Zia H., Quadir A. (2007). Evaluation of Selected Micronized Poloxamers as Tablet Lubricants. Drug Deliv..

[4] Ribet J., Poret K., Arseguel D., Chulia D., Rodriguez F. (2003). Talc Functionality as Lubricant: Texture, Mean Diameter, and Specific Surface Area Influence. Drug Dev. Ind. Pharm..

[5] Colloidal Silicon Dioxide from MilliporeSigma. https://www.americanpharmaceuticalreview.com/25260-Excipients/5821286-Colloidal-Silicon-Dioxide/.

[6] Uzunović A., Vranić E. (2007). Effect of Magnesium Stearate Concentration on Dissolution Properties of Ranitidine Hydrochloride Coated Tablets. Bosn J. Basic Med. Sci..

[7] Paul S., Sun C.C. (2017). Lubrication with Magnesium Stearate Increases Tablet Brittleness. Powder Technol..

[8] Perveen S., Hamid S., Usman S., Hassan S. (2018). In Vitro Dissolution of Metronidazole (400 Mg) Tablets: Effects of Lubricants on The Dissolution of Tablets. Am. J. PharmTech Res..

[9] Sabbatini B., Perinelli D.R., Palmieri G.F., Cespi M., Bonacucina G. (2023). Sodium lauryl sulfate as lubricant in tablets formulations: Is it worth?. Int. J. Pharm..

[10] Yu D., Nie H. (2022). Evaluation of Alternative Metallic Stearates as Lubricants in Pharmaceutical Tablet Formulation. AAPS PharmSciTech.

[11] Turkoglu M., Sahin I., San T. (2005). Evaluation of Hexagonal Boron Nitride as a New Tablet Lubricant. Pharm. Dev. Technol..

[12] Hadinoto K., Tran T.-T., Cheow W.S. (2022). Beyond Tablets’ Physical Characteristics: Incorporating Environmental Sustainability Metrics into the Selection of Lubricants for Pharmaceutical Tableting. J. Clean. Prod..

[13] Gul K., Yousuf B., Singh A.K., Singh P., Wani A. (2015). Rice Bran: Nutritional Values and Its Emerging Potential for Development of Functional Food–A Review. Bioact. Carbohydr. Diet. Fibre.

[14] Lavanya M.N., Saikiran K.C.H.S., Venkatachalapathy N. (2019). Stabilization of Rice Bran Milling Fractions Using Microwave Heating and Its Effect on Storage. J. Food Sci. Technol..

[15] Yılmaz Tuncel N. (2023). Stabilization of Rice Bran: A Review. Foods.

[16] Sapwarobol S., Saphyakhajorn W., Astina J. (2021). Biological Functions and Activities of Rice Bran as a Functional Ingredient: A Review. Nutr. Metab. Insights.

[17] Alonso-González M., Felix M., Romero A. (2022). Rice Bran-Based Bioplastics: Effects of Biopolymer Fractions on Their Mechanical, Functional and Microstructural Properties. Polymers.

[18] Khongla C., Chuaingan J., Siadkhunthod T., Somnam P., Musika S., Sangsawad P. (2022). Physicochemical Properties of Rice Bran Hydrolysate Prepared in a Pilot Scale Process and Its Application in Milk Tablets. Trends Sci..

[19] Pourfarzad A., Yousefi A. (2021). Effect of Different Excipients on Physicochemical Properties of the Functional Rice Bran Tablet: Univariate and Multivariate Studies on a Novel Food Supplement. J. Food Meas. Charact..

[20] Fernández-Ledesma E., Rodríguez-Acosta C., Liva-Garrido M., Díaz-Polanco I., Cazanave-Guarnaluce D. (2015). Evaluation of Rice Husk as an Excipient for the Pharmaceutical Industry. J. Mater. Environ. Sci..

[21] Ribas F.B.T., Gasparetto H., Salau N.P.G. (2023). Sustainable Extraction of Rice Bran Oil: Assessing Renewable Solvents, Kinetics, and Thermodynamics. Chem. Eng. Res. Des..

[22] Abhirami P., Venkatachalapathy N. (2019). Characterization of Refined Rice Bran Wax: An Alternative Edible Coating. Int. J. Curr. Microbiol. Appl. Sci..

[23] Basarkar U.G. (2013). Rice Bran Wax–A Novel Excipient for Pharmaceutical Topical Dosage Forms. Int. J. Bioassays.

[24] Sabale V., Sabale P.M., Lakhotiya C.L. (2009). Comparative Evaluation of Rice Bran Wax as an Ointment Base with Standard Base. Indian J. Pharm. Sci..

[25] Malviya R., Khirsagar M.D., Chandewar A.V. (2017). Studies on Rice Bran Wax as Modified Pharmaceutical Excipient. Int. J. Pharm. Biol. Arch..

[26] Tangkhajornchaisak P., Rojsitthisak P., Tiyaboonchai W., Wattanaarsakit P. (2016). The Effect of Rice Bran Wax on Physicochemical Properties of Curcuminoid-Loaded Solid Lipid Nanoparticles. Thai J. Pharm. Sci..

[27] United States Pharmacopeia USP36/NF31. <401> Fats and Fixed Oils.

[28] Espinales C., Cuesta A., Tapia J., Palacios-Ponce S., Peñas E., Martínez-Villaluenga C., Espinoza A., Cáceres P.J. (2022). The Effect of Stabilized Rice Bran Addition on Physicochemical, Sensory, and Techno-Functional Properties of Bread. Foods.

[29] AOAC (2007). AOAC Official Method 925.19 Loss on drying (moisture) in Tea. AOAC Official Methods of Analysis.

[30] AOAC (2007). AOAC Official Method 942.25 Ash in animal feed. AOAC Official Methods of Analysis.

[31] AOAC (2007). AOAC Official Method 992.23 Crude protein in cereal grains and oilseeds. AOAC Official Methods of Analysis.

[32] AOAC (2007). AOAC Official Method 920.39 Fat (crude) or ether extract in animal feed. AOAC Official Methods of Analysis.

[33] United States Pharmacopeia USP34/NF29. <1174> Powder Flow.

[34] Oliveira R., Oliveira V., Aracava K.K., da Costa Rodrigues C.E. (2012). Effects of the Extraction Conditions on the Yield and Composition of Rice Bran Oil Extracted with Ethanol—A Response Surface Approach. Food Bioprod. Process..

[35] Maru A.D., Surawase R.K., Bodhe P.V. (2012). Studies on Physico-chemical of Rice Bran Wax and Its Comparison with Carnauba Wax. Int. J. Pharm. Phytochem. Res..

[36] Kumari N., Vinita N.K., Rani P. (2018). Nutrient Composition of Full Fat and Defattated Rice Bran. Asian J. Dairy Food Res..

[37] Gravel A., Marciniak A., Couture M., Doyen A. (2021). Effects of Hexane on Protein Profile, Solubility and Foaming Properties of Defatted Proteins Extracted from Tenebrio molitor Larvae. Molecules.

[38] Hsu S.H., Tsai T.R., Chuo W.H., Cham T.M. (1997). Evaluation of Era-Tab as a Direct Compression Excipient. Drug Dev. Ind. Pharm..

[39] De Backere C., Quodbach J., De Beer T., Vervaet C., Vanhoorne V. (2022). Impact of Alternative Lubricants on Process and Tablet Quality for Direct Compression. Int. J. Pharm..

[40] Carpanzano T., Best Practices for Effective Tablet Lubrication: An Executive Summary Pharmaceutical Technology 2017; p. 5. https://www.pharmtech.com/view/best-practices-effective-tablet-lubrication.

